# Potential Inhibitors of Human–*Naegleria fowleri* Interactions: An In Vitro Extracellular Matrix-Based Model

**DOI:** 10.3390/md23080306

**Published:** 2025-07-30

**Authors:** Javier Chao-Pellicer, Iñigo Arberas-Jiménez, Ines Sifaoui, Ana R. Díaz-Marrero, José J. Fernández, Melissa Jamerson, José E. Piñero, Jacob Lorenzo-Morales

**Affiliations:** 1 Instituto Universitario de Enfermedades Tropicales y Salud Pública de Canarias, Universidad de La Laguna, Avda. Astrofísico Fco. Sánchez, S/N, 38203 La Laguna, Tenerife, Islas Canarias, Spain; jchaopel@ull.edu.es (J.C.-P.); iarberas@ull.edu.es (I.A.-J.); isifaoui@ull.edu.es (I.S.); 2 Departamento de Obstetricia y Ginecología, Pediatría, Medicina Preventiva y Salud Pública, Toxicología, Medicina Legal y Forense y Parasitología, Universidad de La Laguna, 38200 La laguna, Tenerife, Islas Canarias, Spain; 3 CIBER de Enfermedades Infecciosas (CIBERINFEC), Instituto de Salud Carlos III, 28029 Madrid, Spain; 4 Instituto Universitario de Bio-Orgánica Antonio González (IUBO AG), Universidad de La Laguna (ULL), Avenida Astrofísico Francisco Sánchez 2, 38206 La Laguna, Tenerife, Spain; adiazmar@ull.edu.es (A.R.D.-M.); jjfercas@ull.edu.es (J.J.F.); 5 Instituto de Productos Naturales y Agrobiología (IPNA), Consejo Superior de Investigaciones Científicas (CSIC), Avenida Astrofísico Francisco Sánchez 3, 38206 La Laguna, Tenerife, Spain; 6 Biotecnología Marina, Unidad Asociada al IPNA-CSIC por el IUBO-ULL, 38206 La Laguna, Spain; 7 Departamento de Química Orgánica, Universidad de La Laguna (ULL), Avenida Astrofísico Francisco Sánchez s/n, 38206 La Laguna, Tenerife, Spain; 8 Department of Microbiology and Immunology, School of Medicine, Virginia Commonwealth University, Richmond, VA 23298, USA; 9 Department of Medical Laboratory Sciences, College of Health Professions, Virginia Commonwealth University, Richmond, VA 23298, USA

**Keywords:** *Naegleria fowleri*, adhesion, laminin, amphotericin B, staurosporine, laurinterol, (+)-elatol

## Abstract

Primary amoebic meningoencephalitis (PAM) is a rapidly progressive and fulminant disease that affects the central nervous system caused by the free-living amoeba *Naegleria fowleri*. The adhesion to extracellular matrix (ECM) proteins is considered as one of the key steps in the success of the infection and could represent an interesting target to be explored in the prevention and treatment of the disease. In this work, the effect of two sesquiterpenes with proven anti-*Naegleria* activity on the adhesion of the parasite was evaluated using an in vitro ECM-based model, compared with the reference drugs amphotericin B and staurosporine. Both laurinterol and (+)-elatol inhibited the adhesion of the *N. fowleri* trophozoites to the main proteins of the ECM when treating them at different concentrations and exposure times. This work not only reinforces the therapeutic potential of laurinterol and (+)-elatol against *N. fowleri* infection but also introduces the application of ECM-based adhesion assays as a novel and valuable tool for screening candidate compounds that disrupt host–pathogen interactions critical to PAM pathogenesis.

## 1. Introduction

The genus *Naegleria* comprises 47 species of free-living amoebae that are distributed ubiquitously worldwide in different aquatic and soil environments. Among them, *N. fowleri* is the only species capable of infecting humans and causing a fatal central nervous system (CNS) disease known as primary amoebic meningoencephalitis (PAM) [1,2]. This protozoan can be isolated from soil and warm freshwater spots that range from 30 to 46 °C, namely rivers, lakes, hot springs, or poorly maintained swimming pools [3].

The infections are more commonly found in children and young adults who performed water-related activities in contaminated locations shortly before manifesting symptom onset. The vegetative stage of *N. fowleri* enters the human when contaminated water is splashed or inhaled into the nasal cavity, allowing the attachment of the amoebae to the olfactory mucosa. Subsequently, trophozoites cross the cribriform plate via the olfactory nerves and reach the olfactory bulbs of the CNS [4,5]. At that point, *N. fowleri* induces an intense immune response that includes neutrophils and macrophages, as well as the release of cytolytic molecules and the presence of food cups that enables the organism to ingest human tissue. These processes lead to CNS tissue damage and hemorrhage, resulting in a fatality rate exceeding 95% [6,7].

**Figure 1:**
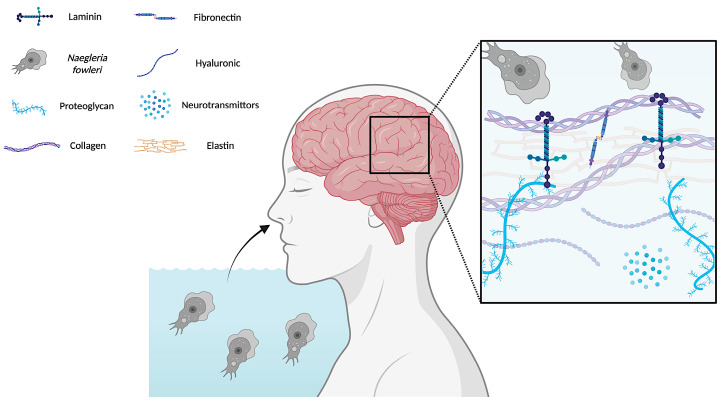
Following inhalation, *Naegleria fowleri* trophozoites migrate towards the brain. The parasite’s surface-binding proteins specifically recognize and attach to various structural components of the ECM. This interaction facilitates the invasion of the ECM, enabling the trophozoites to penetrate deeper into the brain tissue.

The adhesion step plays a pivotal role in the infection process of *N. fowleri*. Moreover, it is considered as one of the most important contact-dependent mechanisms in the pathogenicity of the amoebae since it is a key phase in *N. fowleri*-mediated host cell damage [8]. This process involves different adhesins expressed in the membrane of *N. fowleri*, including integrin-like structures, fibronectin-binding proteins, and C-type protein kinase [8,9]. Specifically, adhesion relies on the binding of the amoebae to extracellular matrix (ECM) proteins, such as laminin-1, collagen I, and fibronectin (Figure 1) [10].

Laminin-1 is an important attachment protein related to cell survival, proliferation increase, or maintenance and induction of the differentiated state [11]. Furthermore, it is also known for being an important ligand for some parasitic protozoa, like *Trichomonas* spp. [12]. Collagen, the most abundant protein in humans, contributes to the maintenance of tissue integrity [13] and is targeted by a wide range of pathogenic protozoa [14]. Fibronectin, a glycoprotein highly represented in the ECM, plays vital roles during tissue repair, cell growth, differentiation, and migration [15,16]. It is also considered an important binding site for diverse microorganisms, including bacteria and protozoa, which represents a major gateway to the host cells of the infected organism [17,18].

In this sense, the role of the ECM in the infection process and its interaction with different pathogenic protozoa has been widely described. For instance, *Leishmania* spp. express high levels of surface metalloprotease gp63, which enhances the capacity of the protozoa to migrate through the ECM and invade host cells [19]. *Trypanosoma cruzi* also has different adhesins that can bind ECM components such as gp 58/68, LAG bp, penetrin, and Ln-binding GP [20]. The ability of *Entamoeba histolytica* to form amoebic invadosomes to facilitate colonic tissue invasion has recently been described. Amoebic EhMSP-1 surface metalloprotease acts on the ECM, leading to the formation of these invasion structures [21].

The study of the adhesion mechanisms of *N. fowleri* has revealed that cellular changes occur during this process in order to bind the fibronectin [9]. Moreover, pathogenic *N. fowleri* exhibits greater affinity for ECM components (laminin-1, collagen I, and fibronectin) than non-pathogenic *N. lovaniensis*. In addition, morphological changes, such as the presence of lamellipodia and focal adhesion-like structures, have been observed when facing *N. fowleri* to ECM proteins, suggesting their role in the pathogenicity of this amoeba [10].

**Figure 2:**
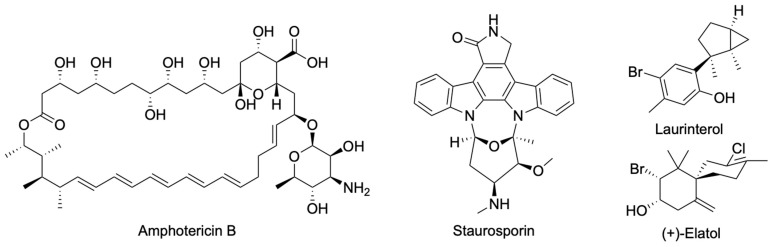
Chemical structure of chemotherapeutic agents against *N. fowleri*: amphotericin B, staurosporin, laurinterol, and (+)-elatol.

Since the adhesion process represents a key step in *N. fowleri* infection and PAM development, it can be considered as a potential target for the treatment of this disease. In this work, the effect of laurinterol and (+)-elatol, two sesquiterpenes with proved anti-*Naegleria* activity [22,23], on *N. fowleri* adhesion was evaluated and compared with the reference drugs amphotericin B and staurosporine (Figure 2).

Additionally, this study constitutes the first report of adhesion assays using ECM proteins to assess the anti-adhesive potential of candidate chemotherapeutic agents against *N. fowleri*. These assays serve as complementary tools to standard 96-well plate viability tests, providing more specific insights into the mechanisms by which compounds may interfere with host–parasite interactions. Alongside laurinterol and (+)-elatol, the experiments included amphotericin B and staurosporine as positive controls due to their known anti-amoebic activity. Amphotericin B, a polyene antifungal agent, remains the most frequently used treatment in clinical cases of PAM [24,25], while staurosporine, a molecule isolated from *Streptomyces*, has demonstrated in vitro activity against *N. fowleri* [26]. Although neither of these agents had been previously evaluated in adhesion-based assays, their inclusion in this study serves as a reference control for evaluating the assay’s performance.

## 2. Results

### 2.1. Laminin Adhesion Assays

**Figure 3:**
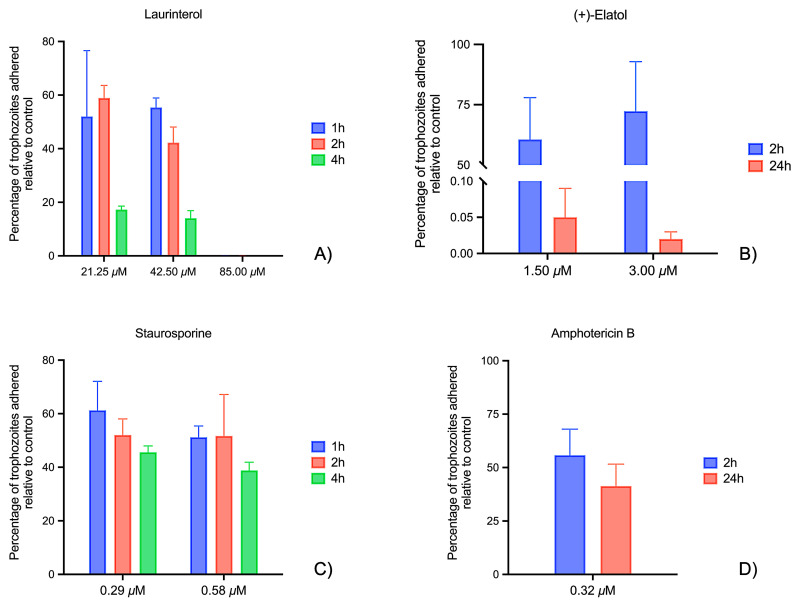
Effect of laurinterol (**A**) and (+)-elatol (**B**) on *Naegleria fowleri* adhesion to laminin. The graph shows the percentage of attached cells at different laurinterol concentrations (85.00, 42.50, and 21.25 μM) over three incubation times (1 h, 2 h, and 4 h), as well as at different (+)-elatol concentrations (3.00 and 1.50 μM) over two incubation times (2 h and 24 h). A time-dependent decrease in adhesion was observed for both compounds. Laurinterol drastically reduced adhesion, particularly at 85.00 μM, where no attachment was detected. In contrast, (+)-elatol had a slower effect, with high adhesion after 2 h but a strong reduction after 24 h. Staurosporine (**C**) and amphotericin B (**D**) were included as reference drugs, showing moderate reduction in adhesion over the tested time points. Data are expressed as mean ± SD.

In Figure 3, the percentage of laminin-attached cells after different treatment conditions in comparison to the negative control (untreated cells) is represented. Laurinterol-treated cells exhibited minimal attachment to laminin at the highest concentration. Moreover, after 4 h of treatment, less than 20% of the amoebae could attach to the protein even at the lowest concentration. In this sense, a significant decrease in the number of attached amoebae was observed between 2 and 4 h of incubation (see Supplementary Material).

In contrast, the effect of (+)-elatol on the trophozoites was slower compared to the laurinterol. After 2 h of treatment, more than 70% of the cells remained attached. A more pronounced effect was observed after 24 h of incubation at both concentrations.

### 2.2. Matrigel^®^ Matrix Adhesion Assays

**Figure 4:**
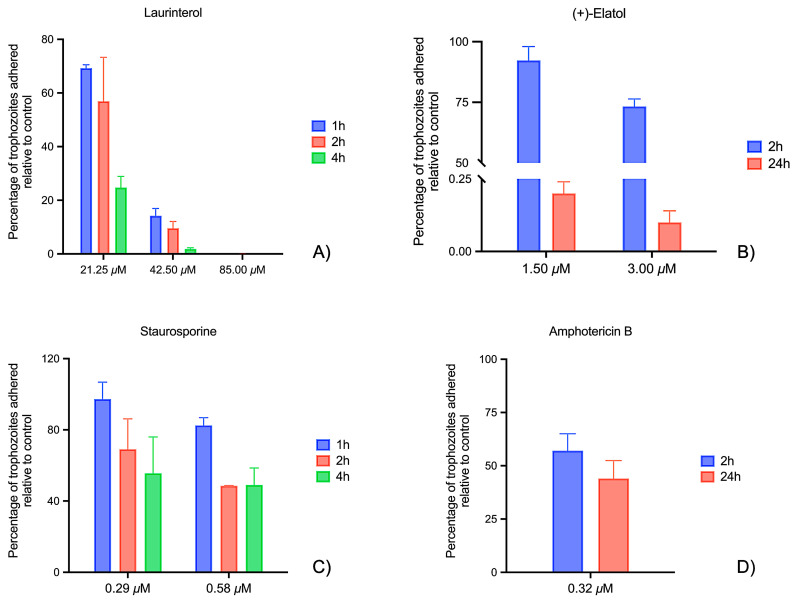
Effect of laurinterol (**A**) and (+)-elatol (**B**) on *Naegleria fowleri* adhesion to Matrigel^®^ matrix. The graph shows the percentage of attached cells at different laurinterol concentrations (85.00, 42.50, and 21.25 μM) over three incubation times (1 h, 2 h, and 4 h), as well as at different (+)-elatol concentrations (3.00 and 1.50 μM) over two incubation times (2 h and 24 h). For laurinterol, a time-dependent decrease in adhesion was observed, with no adhesion detected at 85.00 μM and a reduction at 42.50 μM and 21.25 μM. For (+)-elatol, adhesion remained high at 2 h but dropped significantly after 24 h, with almost no adhesion at both concentrations. Reference compounds staurosporine (**C**) and amphotericin B (**D**) also reduced adhesion over time. Data are expressed as mean ± SD.

The relative percentage of amoeba adhesion compared to the negative control cells is illustrated in Figure 4. As observed in the previous experiment, almost no cells remained attached after treatment with laurinterol at 85.00 µM. At 42.50 µM. A significant difference in the number of adhered cells was observed between 1 and 4 h of treatment. Specifically, the percentage of adhered trophozoites decreased from 55.33 ± 3.58% at 1 h to 14.03 ± 2.83% at 4 h, representing a 74.6% reduction in adhesion. Finally, a significantly higher number of trophozoites were attached to the Matrigel^®^ matrix when exposed to a lower concentration of laurinterol (see Supplementary Material).

For (+)-elatol, more than 70% of the cells remained attached after 2 h of incubation at both tested concentrations. Specifically, at 3.00 µM, the adhesion was 73.23 ± 3.12% after 2 h, but it dropped significantly to 0.20 ± 0.04% after 24 h. At 1.50 µM, the adhesion was even higher, reaching 92.23 ± 5.72% after 2 h, with a substantial decrease to 0.10 ± 0.04% after 24 h.

## 3. Discussion

The extracellular matrix (ECM) proteins collagen I, fibronectin, and laminin-1 are critical targets for the invasive amoeba *Naegleria fowleri*, which is the causative agent of primary amoebic meningoencephalitis (PAM). The amoeba’s ability to attach to these ECM proteins is a pivotal step in its pathogenesis, facilitating infection and enabling its invasion of the central nervous system (CNS). In addition to adhesion, *N. fowleri* secretes diverse proteases, including metalloproteinases and serine proteases, which degrade ECM components and promote the progression of infection [27,28,29]. This mechanism, which is common to other invasive pathogens, may play a central role in the disruption of the blood–brain barrier (BBB) and the subsequent invasion of the CNS [30].

Previous studies by our research group have demonstrated that diverse sesquiterpenes from the red algae *Laurencia* exhibit in vitro activity against *N. fowleri* trophozoites in conventional 96-well plate assays [22,23]. In view of these results, an evaluation of their effects on amebic adhesion to ECM proteins was performed. Considering the critical role of adhesion in *N. fowleri* pathogenicity, two natural sesquiterpenes, (+)-elatol and laurinterol, were evaluated for their inhibitory potential in this process. Initially, the adhesion of the amoebae to the main protein of the ECM, laminin, was performed. Based on the inhibitory effect observed, the study was extended to assess adhesion to an ECM-like membrane. These compounds notably reduced trophozoite attachment to ECM components, suggesting an interference with the adhesion process itself. Our obtained results were compared with those of amphotericin B and staurosporine, both highly active molecules against this pathogen. Amphotericin B exerts its anti-amoebic activity primarily through its interaction with ergosterol-like molecules in the plasma membrane of *N. fowleri*, leading to pore formation, membrane destabilization, and cell lysis [31]. Staurosporine, on the other hand, acts as a broad-spectrum protein kinase inhibitor that can trigger apoptotic-like processes in protozoa by interfering with signaling pathways essential for cell survival and proliferation [26]. Natural compounds such as laurinterol and (+)-elatol exhibit different modes of action. Laurinterol has been suggested to trigger programmed cell death (PCD) by inhibition of ATPases in *N. fowleri* [26], whereas (+)-elatol is a specific inhibitor of ATP hydrolysis by the eukaryotic translation initiation factor 4A (eIF4A1) [31]. These distinct mechanisms contribute to their potent amoebicidal effects and support their use as reference compounds in adhesion-based assays.

The effect of laurinterol was particularly pronounced. At the highest concentration (85.00 µM), this compound caused a substantial reduction in trophozoite attachment to laminin-coated plates within 2 h, with almost complete inhibition observed at later time points. At lower concentrations (42.50 and 21.25 µM), the inhibition was less pronounced but still significant, with a progressive decrease in adhesion over time. Laurinterol also showed a fast reduction in adhesion on Matrigel^®^-coated plates, demonstrating its fast-acting properties. This fast action may be attributed to its interaction with the amoeba’s membrane, though further studies are needed to elucidate its precise mechanism of action.

In contrast, (+)-elatol exhibited a more delayed but sustained inhibitory effect on adhesion. At 3.00 µM, significant inhibition was observed at 24 h (0.02 ± 0.01%), while the effect was less marked at 2 h (72.31 ± 20.56%). Lower concentrations of (+)-elatol (1.50 µM) still showed anti-adhesive activity, albeit with a less pronounced effect. These results indicate a time-dependent response for (+)-elatol. In contrast, laurinterol produced near-complete inhibition within 4 h, and longer incubation times were thus omitted. As a result, while the kinetics differ, both compounds ultimately disrupt amoebic adhesion. The delayed effect of (+)-elatol may be attributed to differences in molecular stability, ECM diffusion, or slower interaction with parasite structures, warranting further investigation. Although PAM is a rapidly progressing disease, evaluating longer incubation periods in vitro enables the detection of compounds with delayed yet sustained anti-adhesive effects, which could be advantageous in clinical scenarios where CNS penetration of the drug may be prolonged.

When compared to reference drugs, both sesquiterpenes exhibited similar or even higher activity at certain concentrations and time points. For instance, amphotericin B (0.32 µM) reduced the attachment by 41.36 ± 10.24% at 24 h, and staurosporine (0.58 µM) maintained a decrease of around 51–52%. At the highest concentrations evaluated, laurinterol (85.00 µM) and (+)-elatol (3.00 µM) showed stronger inhibition, particularly at later time points, underscoring their potential as more effective alternatives to current treatments. Regarding cytotoxicity, (+)-elatol exhibited a CC_50_ of 61.52 ± 12.97 µM [23], while laurinterol showed a CC_50_ of 80.11 ± 7.79 µM [22] in murine macrophage cells J774A.1 (ATCC TIB-67), using a colorimetric assay based on the alamarBlue cell viability method [23,32]. Although less potent than amphotericin B (IC_50_ = 0.12 ± 0.03 µM; CC_50_ > 200 µM) [22] and staurosporine (IC_50_ = 0.08 ± 0.01 µM; CC_50_ = 8.74 ± 0.72 µM) [26], the sesquiterpenes displayed measurable selectivity, particularly in the case of (+)-elatol. These findings highlight the potential of both compounds as scaffolds for further chemical optimization to improve efficacy and reduce host toxicity.

The results from Matrigel^®^ assays mirrored those from laminin, with laurinterol showing a similar reduction in adhesion at earlier time points, and (+)-elatol demonstrating more sustained inhibition over time. These findings emphasize the potential of these compounds in disrupting *N. fowleri* adhesion to ECM proteins, which could provide a novel therapeutic strategy for treating PAM.

In conclusion, laurinterol and (+)-elatol exhibit promising anti-adhesion properties, suggesting their potential as therapeutic agents for PAM infections. Their efficacy, particularly compared to reference drugs like amphotericin B and staurosporine, highlights the value of marine-derived compounds as alternatives to current treatments. However, further research is needed to better understand their mechanisms of action, explore potential synergistic effects with existing therapies, and assess their bioavailability and efficacy in in vivo models. Given the high fatality rate associated with PAM and the limited efficacy of existing treatments, developing alternative therapeutic strategies is critical. Future studies should focus on elucidating the precise molecular targets of these compounds to determine their relevance as potential therapeutics for PAM. Moreover, this work represents the first attempt to evaluate the effect of different molecules on the adhesion of *N. fowleri* to ECM protein-coated wells, becoming a novel tool for the search for new therapies against this deadly parasite. These findings support the potential role of ECM adhesion inhibition as a complementary therapeutic strategy in PAM. While traditional treatments focus on parasite viability, preventing adhesion to host tissues could block the initial stages of infection, reducing the likelihood of CNS entry. Compounds that act on this step may be especially valuable when combined with amoebicidal agents, offering a dual mechanism of action to improve therapeutic efficacy. Furthermore, adhesion-based assays provide an alternative screening platform that may reveal mechanisms of action not captured by viability assays alone.

## 4. Materials and Methods

### 4.1. Cell Cultures

*N. fowleri* trophozoites (KUL strain, ATCC^®^ 30808™) obtained from the American Type Culture Collection were used. Amoebae were axenically cultured in a 2% bactocasitone medium supplemented with 10% (*v*/*v*) of fetal bovine serum (Biowest, VWR, Barcelona, Spain). Additionally, 0.3% penicilin G sodium salt (Sigma-Aldrich, Madrid, Spain), and 0.5 mg/mL streptomycin sulfate (Sigma-Aldrich, Madrid, Spain) were added. The cultures were maintained at 37 °C in an incubator to ensure optimal growth conditions.

### 4.2. Chemicals

The evaluated sesquiterpenes, laurinterol and (+)-elatol, were originally isolated from *Laurencia* spp., obtained from the compound collection of IPNA-CSIC. Amphotericin B (Amresco Inc., Solon, OH, USA) and staurosporine (IUBO-ULL chemolibrary), compounds known for their potent anti-amoebic activity, were used as reference compounds in the assays. All compounds were dissolved in 100% filtered DMSO and stored at −20 °C until use.

### 4.3. Laminin Adhesion Assay Procedure

For these assays, 96-well plates pre-coated with mouse laminin-1 were used (Corning Inc., Corning, NY, USA). Firstly, a known concentration of *N. fowleri* trophozoites (5 × 10^5^ cells/mL) was seeded in each well. After that, the evaluated molecules were added at different concentrations using bactocasitone as dilution medium, and plates were placed at 37 °C. Laurinterol-treated amoebae were incubated for 1, 2, and 4 h, whereas amoebae treated with (+)-elatol were incubated for 2 and 24 h. After the incubation, wells were washed with PBS with the aim to remove unattached trophozoites. CellTiter-Glo^®^ Luminescent Cell Viability Assay reagent (Promega Biotech Ibérica S.L, Madrid, Spain) was then used to determine the number of amoebae that were attached to the laminin. Negative control consisted of cells incubated in bactocasitone medium. Finally, the percentage of attached amoebae after each treatment in relation to the negative control cells was calculated. The concentrations of laurinterol and (+)-elatol employed in this study were selected based on their previously established in vitro activity against *Naegleria fowleri* trophozoites. The selected ranges encompass sub-effective, mid-range, and high-inhibitory concentrations, allowing evaluation of dose-dependent anti-adhesive effects without inducing excessive cytotoxicity that could compromise interpretation of adhesion-specific outcomes. Similarly, amphotericin B and staurosporine were included at concentrations known to produce measurable anti-amoebic effects while preserving assay integrity. Time points were selected based on prior studies showing different onset kinetics: laurinterol acted rapidly within the first hours, while (+)-elatol required longer incubation to exhibit its maximal anti-adhesive effect. For amphotericin B and staurosporine, time points were chosen to reflect their known activity profiles against *N. fowleri*. These time frames enabled comparison of the compounds’ anti-adhesion potential within their respective effective windows.

### 4.4. ECM Adhesion Assay Procedure

In order to reproduce the ECM, the Matrigel^®^ Matrix (Corning Inc., Corning, NY, USA) was used. It is mostly composed of laminin and collagen IV, two of the main elements of the basement membrane [33]. Firstly, the coating of wells with the mentioned matrix was performed. For this, 50 µL of the matrix solution was added to each well and the plates were placed at 37 °C for one hour. Following incubation, excess matrix was carefully removed, and the plates were maintained at 37 °C until use. Once the wells were adequately coated, *Naegleria fowleri* trophozoites were seeded, and the experimental procedure described in the previous section was followed. Although Matrigel^®^ does not fully replicate the ECM composition of human brain tissue, its content of key adhesion-related proteins makes it a suitable alternative for adhesion assays. Future studies will aim to validate these findings using purified ECM components or brain-derived matrix preparations to better approximate physiological conditions.

### 4.5. Statistical Analysis

The data obtained from laurinterol and (+)-elatol treatments on both laminin and Matrigel^®^ Matrix were analyzed using a two-way ANOVA to evaluate the effects of concentration and incubation time. Post hoc multiple comparison tests were applied, using Tukey’s test or Šídák’s test accordingly. Detailed statistical comparisons are provided in the Supplementary Material. All data are presented as the mean ± standard deviation (SD) from three independent experiments. Differences were considered statistically significant when *p* < 0.05. Additionally, the graphs show the percentage of attached cells under different experimental conditions.

## 5. Patents

Nocchi, N.; Fernández, J.J. Díaz-Marrero, A.R.; Lorenzo-Morales, J.; Piñero, J.E.; Arberas Jiménez, I.; Sifaoui, I.; Rizo Liendo, A.P202230496. Use of the (+)-elatol compound for treating or preventing a *Naegleria fowleri* infection. Spain. 6 June 2022. International application: PCT/ES2023/070364; 28 June 2023. Publication no.: WO 2023/237795 A1; Universidad de La Laguna.

## Data Availability

All relevant data supporting the findings of this study are included in the main text. Statistical differences and additional analyses are provided in the Supplementary Material. Further inquiries can be directed to the corresponding authors.

## Supplementary Materials

The following supporting information can be downloaded at: https://www.mdpi.com/article/10.3390/md23080306/s1.

## Abbreviations

The following abbreviations are used in this manuscript:

ANOVA	Analysis of variance
BBB	Blood–brain barrier
CNS	Central nervous system
ECM	Extracellular matrix
FLA	Free living amoebae
PAM	Primary amoebic meningoencephalitis
PCD	Programmed cell death
SD	Standard deviation
